# White Cord Syndrome Following Cervical Surgery in a Patient With Klippel-Feil Syndrome: A Case Report

**DOI:** 10.7759/cureus.55353

**Published:** 2024-03-01

**Authors:** Ioannis Chatzikomninos, Eleni Pappa, Christos P Zafeiris, Konstantinos Zygogiannis, Spyridon I Antonopoulos, Ioannis Angelos Trantos, Fotios Kakridonis, Emmanouil Tsafantakis

**Affiliations:** 1 Spine and Scoliosis Department, KAT General Hospital, Athens, GRC; 2 5th Orthopaedic Department, KAT General Hospital, Athens, GRC; 3 Department of Orthopaedics, Metropolitan General Hospital, Athens, GRC; 4 Department of Trauma and Orthopaedics, Laiko General Hospital of Athens, Athens, GRC; 5 Orthopaedic Department, KAT General Hospital, Athens, GRC; 6 2nd Orthopaedic Department, Agia Sofia Children's Hospital, Athens, GRC; 7 Department of Spine Surgery, Metropolitan General Hospital, Athens, GRC

**Keywords:** klippel-feil syndrome, quadriplegia, white cord syndrome, cervical spine stenosis, klippel, cervical spine

## Abstract

White cord syndrome is a rare entity, as there are very few cases described in the current literature. Postoperative MRI examination reveals cord intrinsic changes, including edema and ischemia. It is also described as a reperfusion injury of the spinal cord. This report depicts a rare case of “white cord syndrome” with tetraplegia after posterior laminectomy and fusion of the cervical spine in a patient with Klippel-Feil syndrome. A 33-year-old male patient with Klippel-Feil syndrome presented to our department with cervical myelopathy, claudication, deteriorating neurological status, imbalance, and lower limb spasticity. Due to kyphotic malformation of the cervical spine, a two-stage surgical intervention was scheduled. The patient first underwent anterior spinal fusion of C4-C6 with corpectomy of C5, where many anatomical and visceral differentiations were signed, so the surgical team was enhanced by a vascular surgeon. The postoperative period was uneventful and the patient was discharged after a week of hospitalization without any neurological deterioration. A second surgical intervention was scheduled after two months where laminectomy of C5-C7 and posterior fusion of C5-T1 were carried out. However, due to intraoperative spinal instability and various anatomical spinal variations, a third surgery, which would be occipitocervical fusion, was decided as the final surgical solution. During the third surgical operation, after the laminectomy of C1 to C5 and the placement of the occipital plate, the screws, and the two rods in situ, complete nullification of the intraoperative neurophysiologic control was signed. The internal fixation was removed immediately, the wake-up test revealed tetraplegia below C5, and the patient was transferred to the ICU. Immediate MRI revealed no spinal cord hematoma; however, spinal cord edema was present. The patient underwent a tracheostomy and remained quadriplegic with a sensory level of T8 and motor level of C5 and was discharged to a rehabilitation center. The possibility of white cord syndrome should be explained by surgeons before any cervical decompression surgery, as well as a thorough neurological examination should be performed postoperatively. The early recognition and prompt management of white cord syndrome is recommended.

## Introduction

Anterior and posterior cervical fusion and laminectomy are common procedures in cervical surgery, which are accompanied by good clinical results. However, the most serious complication in cervical surgery is postoperative neurological impairment, including paraplegia and even quadriplegia. However, when a neurological impairment cannot be explained intraoperatively, it is likely to be caused by reperfusion injury, which is also called “white cord syndrome.” White cord syndrome is an extremely rare entity in the current literature, with very few cases described. The common MRI findings include hyperintense cord signals, as well as ischemia and edema. The pathophysiologic mechanism is likely to be reperfusion injury; however, free radical oxygen plays a leading role in harming the spinal cord. We report a case of this complication in a patient with Klippel-Feil syndrome undergoing cervical surgery due to cervical myelopathy [1].

## Case presentation

**Figure 1:**
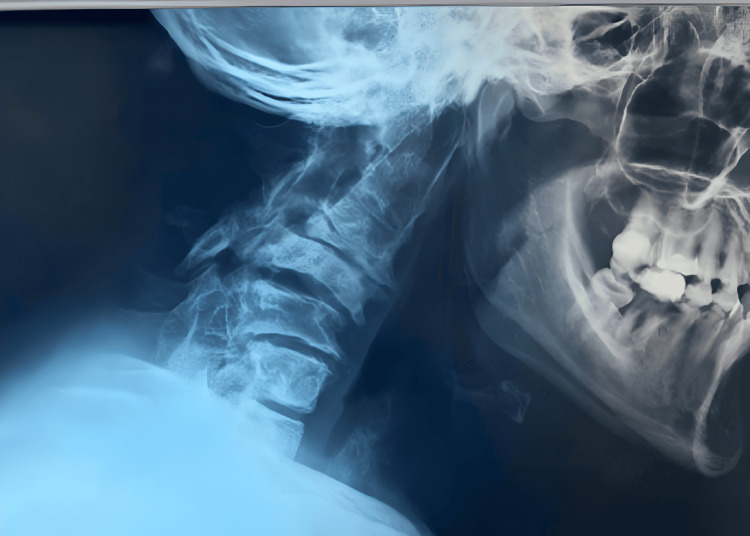
Preoperative lateral view of radiograph of the cervical spine.

**Figure 2:**
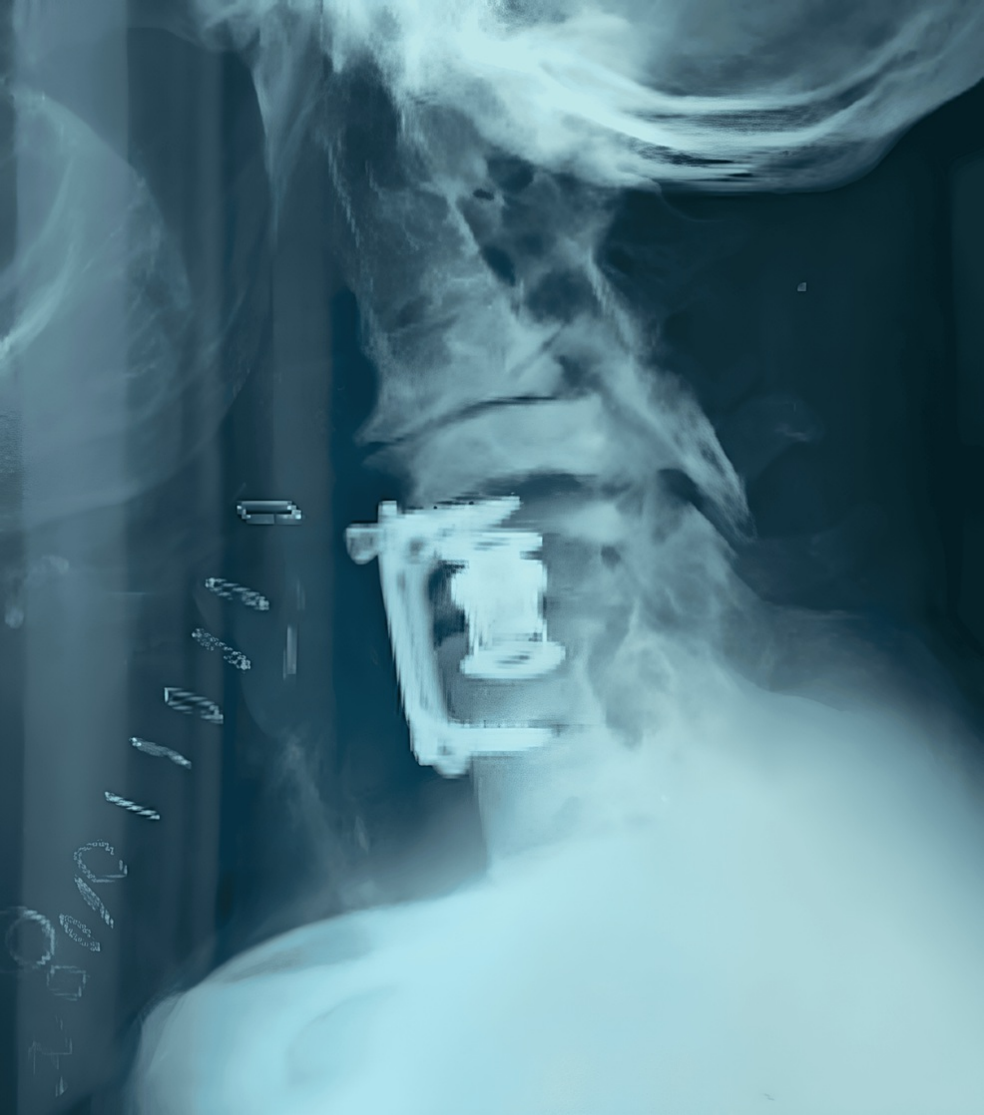
Postoperative lateral view of the radiograph of the cervical spine after the first operation (C4-C7 anterior spinal fusion and corpectomy of C5).

**Figure 3:**
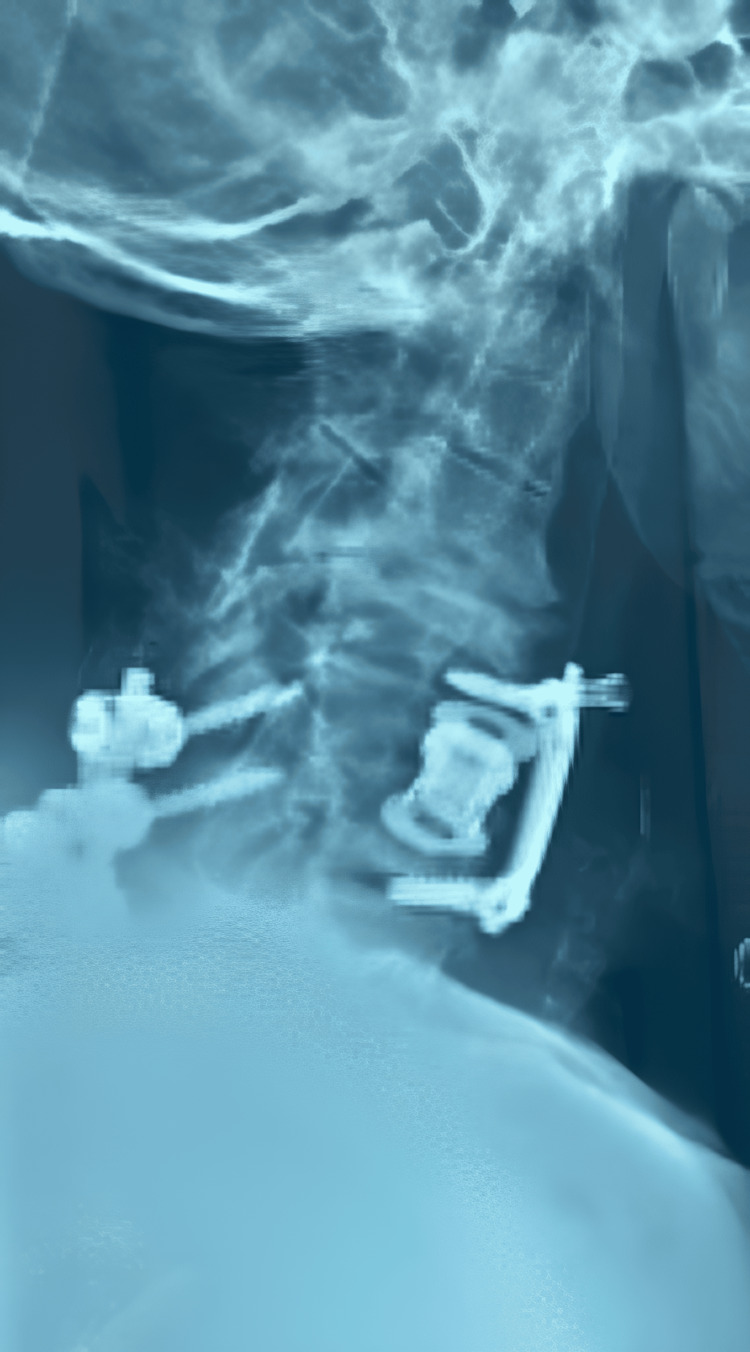
Postoperative lateral view of the radiograph of the cervical spine after the second operation (laminectomy of C5-C7 and posterior fusion of C5-T1).

A 33-year-old male patient with Klippel-Feil syndrome presented to our department with cervical myelopathy, claudication, deteriorating neurological status, imbalance, and lower limb spasticity. Due to kyphotic malformation of the cervical spine, a two-stage surgical intervention was scheduled. The patient first underwent anterior spinal fusion of C4-C6 with corpectomy of C5, where many anatomical and visceral differentiations were signed, so the surgical team was enhanced by a vascular surgeon (Figures 1-3).

**Figure 4:**
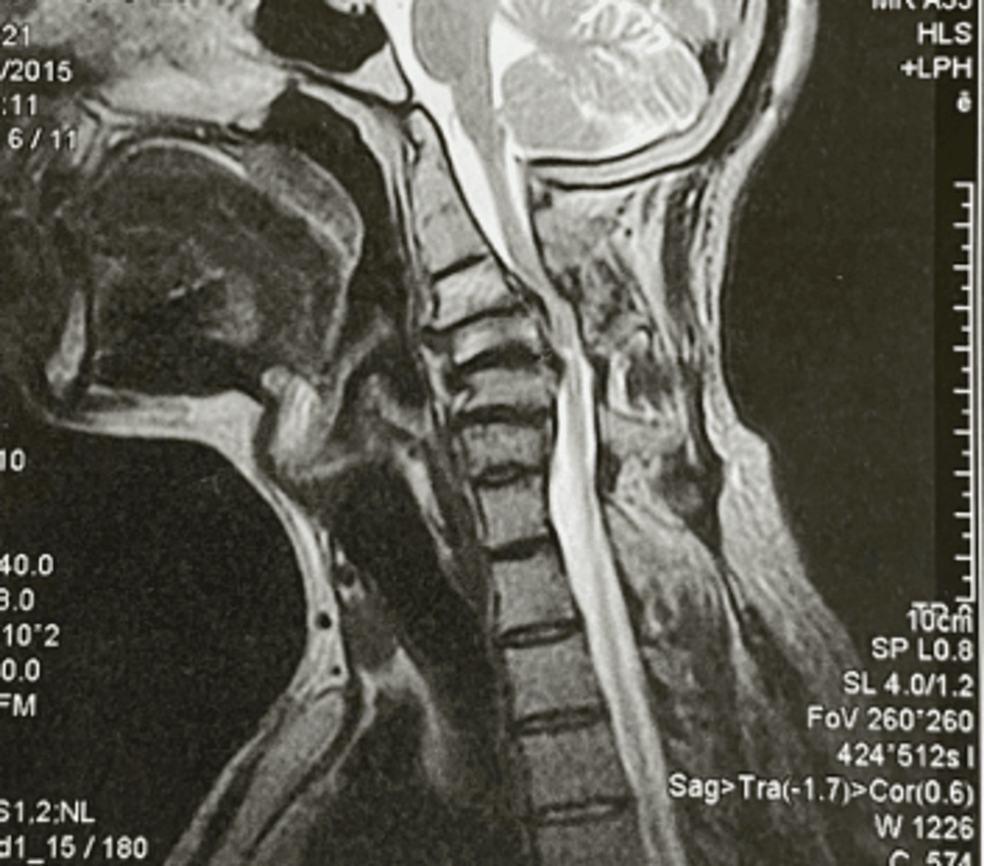
Preoperative MRI of the cervical spine with spinal cord compression present.

**Figure 5:**
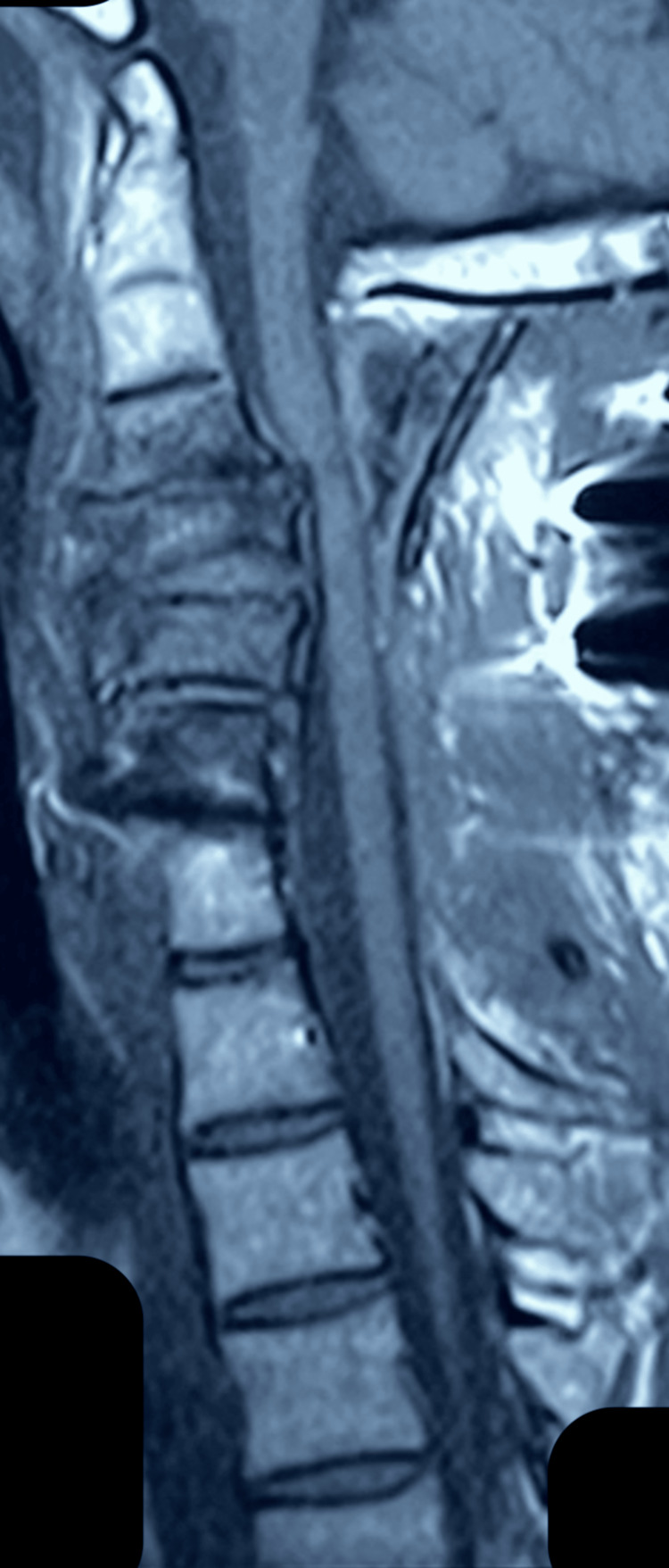
Postoperative MRI of the cervical spine after the third operation (removal of the intraspinal devices).

The postoperative period was uneventful and the patient was discharged after a week of hospitalization without any neurological deterioration. A second surgical intervention was scheduled after two months where laminectomy of C5-C7 and posterior fusion of C5-T1 were carried out. However, due to intraoperative spinal instability and various anatomical spinal variations, a third surgery, which would be occipitocervical fusion, was decided as the final surgical solution. During the third surgical operation, after the laminectomy of C1 to C5 and the placement of the occipital plate, the screws, and the two rods in situ, complete nullification of the intraoperative neurophysiologic control was signed. The internal fixation was removed immediately, intravenous methylprednisolone and mannitol were started and the wake-up test revealed tetraplegia below C5. The patient was transferred to the ICU. Immediate postoperative MRI revealed no spinal cord hematoma; however, spinal cord edema was present as well as a high intrinsic intensity signal within the spinal cord. The patient underwent a tracheostomy and remained quadriplegic with a sensory level of T8 and a motor level of C5. In the last postoperative MRI, a satisfying spinal decompression was present; however, the intrinsic signal of spinal cord myelopathy was the same as preoperatively (Figures 4, 5). Before discharge, both the motor power and sensory deficit did not recover, so the patient was transferred two months postoperatively to a rehabilitation center for further assistance.

## Discussion

White cord syndrome is extremely rare in the current literature. Giamalva et al. reported a case of a 64-year-old man with severe C3-C4 compression who underwent anterior cervical discectomy and fusion (ACDF) of C3-C4 and C5-C6. Immediately postoperatively, the patient suffered tetraparesis, so a high-dose steroid protocol was initiated with partial improvement of the patient [2,3]. Jun et al. also described a case of a 49-year-old woman who underwent ACDF of C6-C7 and suffered from immediate postoperative paraplegia. The postoperative MRI findings included high intrinsic spinal cord signal and edema, with the diagnosis of white cord syndrome having a leading role. The patient underwent laminoplasty and a high-dose steroid protocol, leading to the gradual resolution of the motor power and sensory deficits of both limbs [4].

To our knowledge, this was the first white cord syndrome case caused by posterior cervical operation in a patient with Klippel-Feil syndrome. Quadriplegia occurred in the patient after surgery, so the patient was immediately started on high-dose steroids and all the internal fixation devices were removed, as laminectomy had already been carried out. The exact mechanism of the pathophysiology of white cord syndrome has not been described yet, despite the fact that it is likely to be a reperfusion injury on chronic ischemic tissue due to free radical oxygen molecules [5-7].

In our case, the patient suffered from cervical compression, and the result of cervical decompression led to unexpected neurological deficits. The postoperative MRI findings included hyperintensity and cord edema on the T2 sequence. Despite cervical decompression, removal of the cervical internal devices, and high-dose steroid protocol, the patient did not manage to overcome the motor and sensory deficits. The surgical team was convinced that intraoperatively no injury was made, so we could suggest that the only mechanism that would be able to lead to that neurological deficit would be a reperfusion injury due to oxidative stress [8].

## Conclusions

To conclude, both the extensive decompression and the high-dose steroid protocols in postoperative white cord syndrome are controversial, as no standardized protocols have been mentioned in the current literature. However, they stand adequately as assistive symptomatic treatments. A thorough explanation and warning of the possibility of complications such as white cord syndrome before surgery must be made by the surgeons. Moreover, a detailed neurological examination is essential in every patient with severe cord compression. Finally, the early recognition and treatment of white cord syndrome is highly recommended, so the orthopedic spine surgeon must be suspicious of that entity.
